# Pepino (*Solanum muricatum*) planting increased diversity and abundance of bacterial communities in karst area

**DOI:** 10.1038/srep21938

**Published:** 2016-02-23

**Authors:** Jinxiang Hu, Hui Yang, Xiaohua Long, Zhaopu Liu, Zed Rengel

**Affiliations:** 1 Jiangsu Provincial Key Laboratory of Marine Biology, College of Resources and Environmental Sciences, Nanjing Agricultural University, Nanjing 210095, P.R. China; 2 Soil Science and Plant Nutrition, School of Earth and Environment, The University of Western Australia, 35 Stirling Highway, Crawley WA 6009, Australia

## Abstract

Soil nutrients and microbial communities are the two key factors in revegetation of barren environments. Ecological stoichiometry plays an important role in ecosystem function and limitation, but the relationships between above- and belowground stoichiometry and the bacterial communities in a typical karst region are poorly understood. We used pepino (*Solanum muricatum*) to examine the stoichiometric traits between soil and foliage, and determine diversity and abundance of bacteria in the karst soil. The soil had a relatively high pH, low fertility, and coarse texture. Foliar N:P ratio and the correlations with soil nitrogen and phosphorus suggested nitrogen limitation. The planting of pepino increased soil urease activity and decreased catalase activity. Higher diversity of bacteria was determined in the pepino rhizosphere than bulk soil using a next-generation, Illumina-based sequencing approach. *Proteobacteria*, *Acidobacteria*, *Actinobacteria* and *Bacteroidetes* were the dominant phyla in all samples, accounting for more than 80% of the reads. On a genus level, all 625 detected genera were found in all rhizosphere and bulk soils, and 63 genera showed significant differences among samples. Higher Shannon and Chao 1 indices in the rhizosphere than bulk soil indicated that planting of pepino increased diversity and abundance of bacterial communities in karst area.

The karst landscape degeneration caused by human activities poses restoration challenges as well as opportunities to study the stability and resilience of limestone ecosystems^1^. The karst area of southwest China covers 5.5 × 10^5^ km^2^, with Yunnan province in southwest of China having 6.1 × 10^4^ km^2^ of karst^2^. Karst rocky desertification is a process of land degradation involving serious soil erosion, extensive exposure of basement rocks, drastic decrease in soil productivity, and the appearance of a desert-like landscape^3^. This process was difficult to reverse because the instability and low productivity of such ecosystems weaken soil functions of nutrient cycling^4^,^5^.

Ecological stoichiometry concerns the balance of the multiple chemical elements in ecological interactions^6^. Carbon (C), nitrogen (N) and phosphorus (P) are essential nutrients for all organisms^7^. Nutrient limitation exists widely in terrestrial ecosystems, with N and P limitation being common either individually or together^8^,^9^,^10^,^11^. Foliar N:P ratio has been suggested as an indicator of the nutrient limitation in ecosystems^10^. Previous studies proposed that N:P ratios lower than 14 indicated N limitation, those greater than 16 suggested P limitation, and some transitional states might exist when N:P ratios were between 14 and 16^8^,^12^.

Pepino fruit (*Solanum muricatum*), also known as melon pear and sweet cucumber, is native to South America, but is cultivated in many tropical and subtropical regions^13^,^14^, it was brought to China in 1990 s. It contains more K (>1000 mg kg^−1^), vitamin C (>200 mg kg^−1^) and selenium than most common fruits. In addition, some medicinal properties such as decreasing blood pressure and the diuretic and antitumor activity have been attributed to pepino, potentially increasing its value^15^,^16^,^17^,^18^.

The above- and belowground components of ecosystems are closely correlated, influencing ecosystem processes and properties^19^,^20^. Plants provide substrates to soil organisms in the form of litter and root exudates, and nutrients are supplied to plants by decomposer organisms in soil^21^,^22^,^23^,^24^. Soil enzymes are secreted by microorganisms, animals and roots. These enzymes are major regulators of soil biological processes^25^. Even though soil microorganisms play an important role in belowground ecosystems influencing plant growth and soil properties, the full characterization of soil microbial communities is still a major task, especially in karst regions.

Plants have an important effect on diversity and function of soil microbial communities^26^,^27^,^28^. Bacterial diversity in soils has been studied based on amplified ribosomal DNA restriction analysis (ARDRA)^29^, denaturing gradient gel electrophoresis (DGGE)^30^, phospholipid fatty acid (PLFA)^31^ analyses and so on. Soil nutrients and microbial communities are the two key factors in supporting productivity of ecosystems. In this paper, we focused on the soil remediation processes brought about by pepino planting in Yunnan karst area. We examined the stoichiometry between soil and foliar N:P ratios and characterized bacterial communities in the rhizosphere and non-rhizosphere soil to shed light on the changes caused by pepino planting. We hypothesized that increased soil total nitrogen would enhance soil remediation processes in the karst ecosystem.

## Methods

### Site description

The experimental station is located in Shilin Yi Autonomous County (24°50′N, 103°37′E), Yunnan Province, China. This area is in the centre of Yunnan Plateau with the altitude ranging from 1600 to 2100 m, and has the typical characteristic of karst landform. The annual sunshine is 2079 h. A subtropical mountainous monsoon climate dominates the area with a mean annual temperature of 16.1 °C and a mean annual rainfall of 953 mm. The rainy season is from May to late October, comprising more than 80% of the total rainfall, and the dry season is during autumn and winter. The study area is short of land suitable for cultivation and of surface water resources, and is one of the poorest areas in Shilin County.

In the study area, three 5 × 5 m permanent quadrats (named T1, T2 and T3) were established in the early spring of 2014, each quadrat had three replications. The positions were: T1 (24°50.456′N, 103°37.327′E), T2 (24°50.331′N, 103°37.541′E) and T3 (24°51.058′N, 103°36.439′E). Quadrats T1 and T2 were situated in a natural karst landform unit, and quadrat T3 was selected in the unused agricultural field without karst landform. Each quadrat was positioned using Global Positioning System (GPS) (gpsmap 62S, Garmin Ltd. Beijing, China) to ensure the consistency of terrain and avoid the influence of slope and elevation.

### Sample collection

In the winter of 2013, the soil of three quadrats was ploughed using a modified moldboard plough and then tilled twice prior to planting. In May 2014, pepino shoots were planted in quadrats T2 and T3. Even though the same soil cultivation was also done in quadrat T1, no pepino was planted there.

Before cultivation, three replicate soil samples from depth of 0~10 cm were collected from each quadrat, then kept in zip-lock bags and transported on ice to the laboratory for basic soil analyses. In September 2014, three replicate whole plants were extracted from each quadrat according to Riley and Barber^32^,^33^. The bulk soil (named S2 and S3 for quadrats T2 and T3, respectively) was collected by gently shaking roots, and the rhizosphere soil (named RS2 and RS3 for quadrats T2 and T3, respectively) was collected from soil that adhered to roots. The soil of quadrat T1 (named S1) was collected from the 0–10 cm layer because pepino roots were mainly concentrated in this layer in quadrats T2 and T3. All soil samples were kept in zip-lock bags and transferred on ice to the laboratory. One part of each soil sample was stored at −20 °C for biological and biochemical analyses and the other was air-dried at room temperature one week for chemical analysis.

Plants were divided into different tissues (roots, stalks, leaves and fruits) by scissors and were dried in a forced-air oven at 80 °C for 72 h. All tissues were pulverized using a micromill, passed through a 2-mm sieve and kept dry until chemical analysis.

### Sample analyses

#### Chemical analyses

The analysis of basic chemical properties was done following the published procedure^34^. Soil pH was measured in 1:5 (w/w) soil: CO_2_-free distilled water suspension. Soil and plant tissue organic carbon (C_org_) was determined by oxidation with K_2_Cr_2_O_7_ in a heated oil bath. Total nitrogen (TN) was measured with the semi-micro Kjeldahl method. Total phosphorus (TP) was determined with molybdenum antimony blue colorimetry^35^ after digestion with mixture of perchloric and sulfuric acids. Soil particle composition was measured by laser diffraction using a Mastersizer2000^36^ (Malvern Instruments, Malvern, England). Soil was subjected to microwave digestion, and plant tissues were digested by sulfuric acid and hydrogen peroxide; total Ca^2+^, K^+^, Na^+^ and Mg^2+^ concentrations in soil and plant samples were measured using an inductively coupled plasma atomic emission spectrometer (ICP-AES, Optima 2100DV, Perkin-Elmer, USA)^37^.

#### Biochemical analyses

Soil enzyme activity: The activities of three enzymes (invertase, urease and catalase) were measured. Invertase activity was determined by the colorimetric determination of reducing sugars that reacted with 3,5-dinitrosalicylic acid upon incubation of soil in buffered (0.17 M modified universal buffer, pH 5.5) sucrose solution and toluene at 37 °C for 24 h^38^. Urease activity was detected using improved sodium phenate and sodium hypochlorite colorimetry^39^. Catalase activity determination was based on the recovery rates of H_2_O_2_, and the residual H_2_O_2_ was titrated with KMnO_4_ in the presence of H_2_SO_4_^40^,^41^.

DNA extraction and soil microorganism communities: Three replicate samples were randomly picked from each quadrat and used for DNA extraction. Soil DNA was extracted from 0.30 g of soil (after sieving through a 1-mm mesh) using a PowerSoil DNA Isolation Kit for soil (MO BIO Laboratories, Inc, Carlsbad, CA) according to the manufacturer’s instructions. The extracted soil DNA was dissolved in 100 mL TE buffer (Tris-hydrochloride buffer, pH 8.0, containing 1.0 mM EDTA), quantified by ND1000 and stored at −80 °C before using^42^.

Primers 577F (5′-AYTGGGYDTAAAGNG-3′) and 926R (5′-CCGTCAATTCMTTTRAGT-3′) targeting the regions (V3-V4) of the 16S rRNA gene were used for PCR because sequences in that regions provided the greatest diversity at the domain and bacteria phylum levels^43^. Amplification reactions were performed in 25-μL volume containing 12.5 μL Premix Ex TaqTM Hot Start Version (Takara Biotechnology Co. Ltd, Dalian, China), 0.1 μM of each primer, and 20 ng of template. Amplification was initiated at 98 °C for 3 s, followed by 35 cycles of denaturation at 98 °C for 10 s, primer annealing at 54 °C for 30 s, extension at 72 °C for 45 s, and final extension for 10 min. Amplicon pyrosequencing was performed on an Illumina MiSeq platform at LC-Bio Technology Co., Ltd, Hangzhou, Zhejiang Province, China.

The reads were filtered by QIIME (Quantitative Insights Into Microbial Ecology, http://qiime.org/tutorials/processing_illumina_data.html) quality filters. The CD-HIT pipeline was used for picking operational taxonomic units (OTUs) through making OTU table. Sequences were assigned to OTUs at 97% similarity. Representative sequences were chosen for each OTU, and taxonomic data were then assigned to each representative sequence using the RDP (Ribosomal Database Project) classifier^44^. In order to estimate Alpha Diversity, the OTU table was rarified, and four metrics were calculated: Chao 1 metric to estimate the richness, the Observed OTUs metric as the count of unique OTUs found in the sample, Shannon index and Simpson index^45^.

The complete data sets were deposited in the NCBI, and the GenBank accession numbers are KT784824 - KT792652.

#### Data analyses

All measurements were replicated thrice. The mean values and standard error of all parameters were taken from three replicates. The data were subjected to analysis of variance by one-way ANOVA and Spearman’s rank correlations using SPSS Statistics 19.0 (IBM, Armonk, New York, USA). Individual means were compared using the least significant difference test at the 5% significance level.

## Results

### Soil basic properties and stoichiometric traits

The basic properties of each quadrat before cultivation were presented in Table 1. T1 and T2 quadrats had a relatively high pH (as expected for the limestone ecosystem), and T3 had a significantly lower pH. Compared with T1 and T2, soil in T3 had a significantly lower proportion of sand and higher proportion of silt and clay, suggesting a good texture for plant growth. Influenced by soil erosion and extensive exposure of basement rocks in karst desertification, T1 and T2 had low soil fertility with significantly lower soil organic carbon, total nitrogen and total phosphorus compared with T3.

There was a significant positive correlation between total N and total P or K in karst soils across quadrats T1 and T2 (Table 2). The correlation between total soil K and Ca was negative. Regarding the ratios, organic C was very influential, making the correlation between C:N and C:P significantly positive.

### Relationships between foliar N:P ratios and soil N and P concentrations

**Figure 1:**
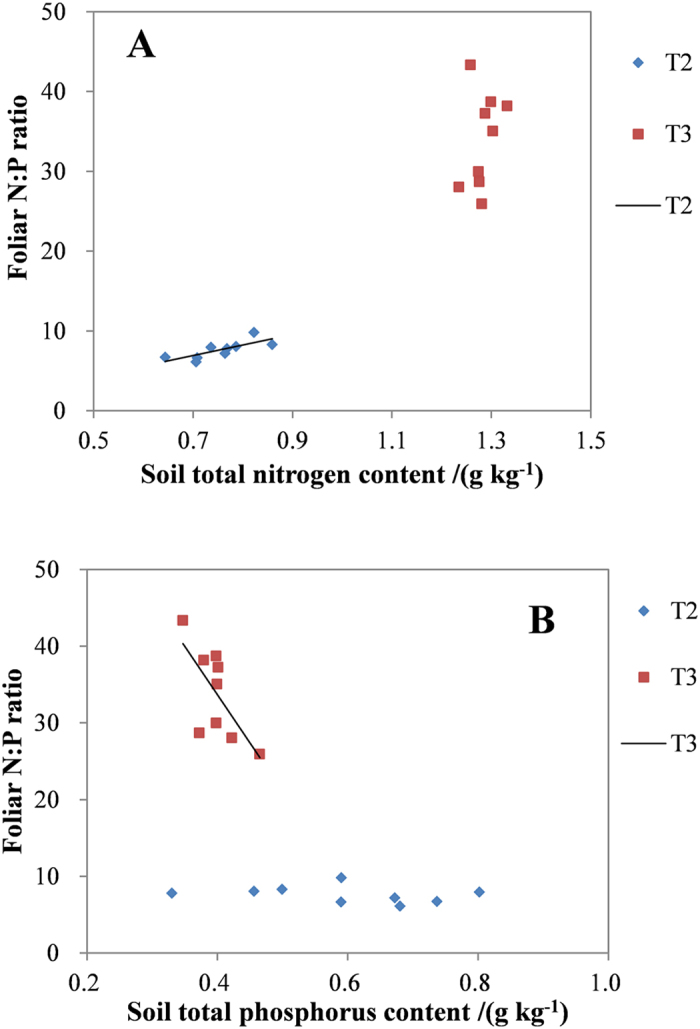
Correlations between foliar N:P ratio and soil total N (**A**) and total P (**B**) for the two quadrats. For (**A**), linear regression was fitted for the T2 (R^2^ = 0.60, p < 0.01, n = 9). For (**B**), linear regression was fitted for T3 (R^2^ = 0.49, p = 0.04, n = 9).

The correlations between foliar N:P ratio and soil total N and total P for the two quadrats were presented in Fig. 1. When each quadrat was analyzed separately, the foliar N:P ratio was positively correlated with soil total N (Fig. 1A) in T2 and was negatively correlated with soil total P (Fig. 1B) in T3.

### Soil enzyme activity

Soil urease activity was highest in S3 and lowest in S1, with S2 and RS2 being significantly higher than S1, but there was no significant difference between S2 and RS2 (Table 3). Catalase activity was highest in S1 followed by S2, and there was a significant difference between S2 and RS2. Invertase activity was highest in RS3 followed by S3, and was significantly higher than RS2, S2 and S1. There was no significant difference between RS2, S2 and S1.

### Soil microbial communities

#### Richness

**Figure 2:**
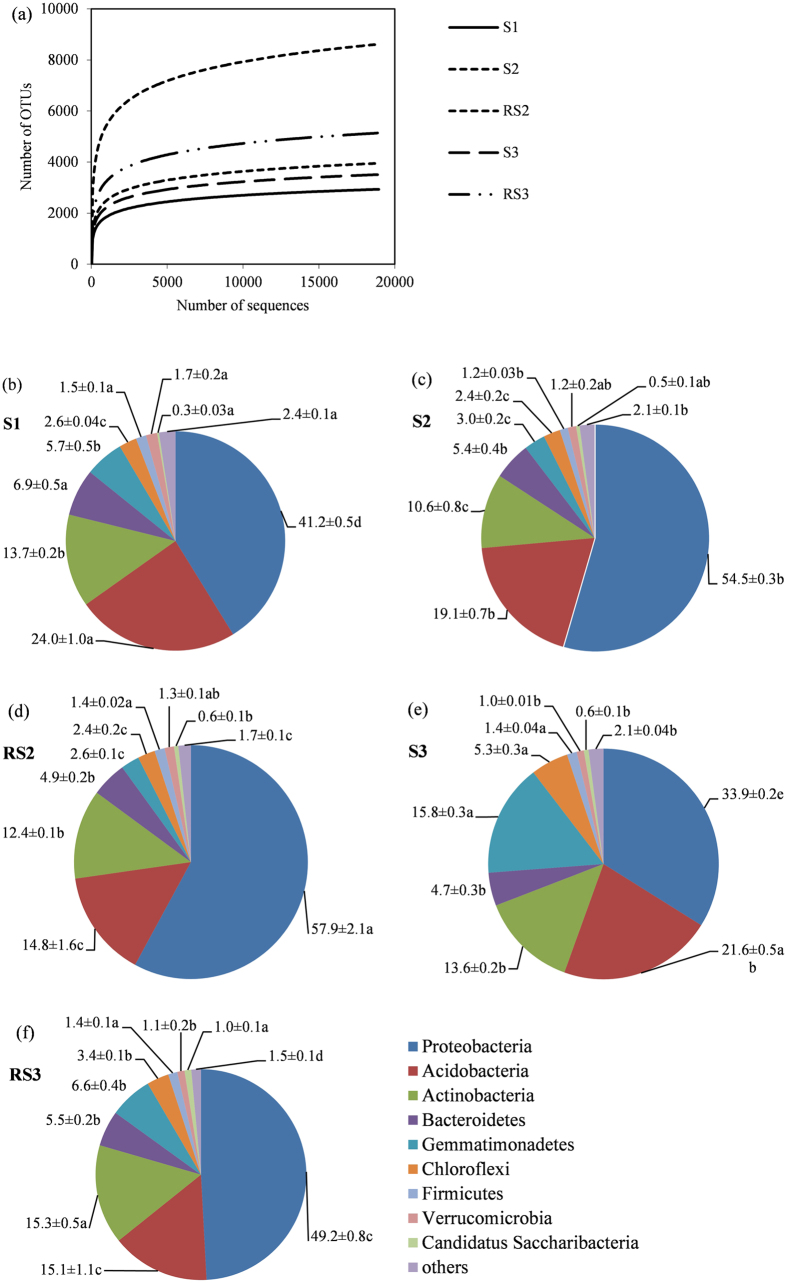
(**a**) Rarefaction curves showing the observed OTU richness (97% identity) of the 16S rRNA gene with increasing sequencing depth. (**b**–**f**) Percentage of different bacteria phyla in each soil sample. Data are means ± standard error (n = 3). One-way ANOVA followed by Duncan test (p = 0.05) was done for each bacterial phylum separately. Sequences that could not be classified into any known groups were labeled “Others”.

More than 10,000 valid reads were selected from each replicate by a sequence optimization process, and the richness indices of bacterial communities were measured (Table 4). A median sequence length of each read was 100 bp after quality filtering. Compared with S1, more than 1,000 additional OTUs were observed in bulk soils S2 and S3 (Fig. 2a).

The rhizosphere soil in quadrat T2 (RS2) had the higher Shannon and Chao 1 indices than the corresponding bulk soil (S2), indicating that the growth of pepino increased diversity of soil bacterial communities (RS2 had the highest Shannon and Chao 1 indices of all soils) (Table 4). Similar results were obtained in comparison between the bulk and rhizosphere soils in T3 (agricultural quadrat), albeit to a slightly lesser degree than in natural T2 soil. The Simpson index was reverse of diversity (the lower the index, the greater biodiversity), and the lowest Simpson index in RS2 confirmed the results derived from the Shannon and Chao 1 indices.

#### Taxonomic coverage

The sequences obtained were distributed in 29 bacterial phyla by RDP (Ribosomal Database Project: http://rdp.cme.msu.edu/index.jsp). In general, bacterial composition of different samples was similar regarding the phyla present, but varied in the distribution of each phylum (Fig. 2b–f). *Proteobacteria*, *Acidobacteria*, *Actinobacteria* and *Bacteroidetes* were the dominant phyla in all samples, accounting for more than 80% of the reads. Compared with S1 and S3 samples, S2 had a significantly higher percentage of *Proteobacteria* (1.3 and 1.6 times, respectively), and lower percentages of *Acidobacteria* (1.3 and 1.1 times, respectively), *Actinobacteria* (1.3 times in both cases), *Gemmatimonadetes* (1.9 and 5.2 times, respectively), *Chloroflexi* (1.1 and 2.2 times, respectively), and *Firmicutes* (1.3 and 1.2 times, respectively). Compared with RS3 sample, RS2 had a significantly higher percentage of *Proteobacteria* (1.2 times), and lower percentages of *Actinobacteria* (1.2 times), *Gemmatimonadetes* (2.6 times), *Chloroflexi* (1.4 times), and *Candidatus Saccharibacteria* (1.7 times). The percentages of *Bacteroidetes* and *Verrucomicrobia* were similar in all samples.

On a genus level, all 625 detected genera were found in all five samples, except for *Escherichia/Shigella*, which were not detected in RS2, *Rhodanobacter* not in S1, and *Burkholderia* not in S2.

The 63 genera with significant differences among samples were listed in Table 5. *Gp6*, *Sphingosinicella*, *Gp16*, *Gaiella*, *Phaselicystis*, *Aciditer*, *Noviherbaspirillum*, *Iamia*, *Ilumatobacter*, *Rhizobium*, *Nitrospira*, *Ottowia*, *Gp17*, and *Parasegetibacter* were significantly higher in S2 compared with RS2, whereas *Pseudolabrys*, *Chondromyces*, *Stakelama*, *Blastomonas*, *Aquincola*, *Gp7*, *Longilinea*, *Novosphingobium*, *Croceicoccus*, *Sphingobium*, and *Devosia* were significantly higher in RS2 compared with S2.

*Sphingosinicella*, *Dongia*, *Ohtaekwangia*, *Steroidobacter*, *Pseudolabrys*, *Phenylobacterium*, *Stakelama*, *Blastomonas*, *Gp10*, *Aquincola*, *Pimelobacter*, *Vasilyevaea*, *Novosphingobium*, *Croceicoccus*, *Ilumatobacter*, *Rhizobium*, *Sphingobium*, *Anderseniella*, *Blastobacter*, *Altererythrobacter*, and *Devosia* were significantly higher in quadrat T2 compared with T1, whereas *Gemmatimonas*, *Gp4*, *Kofleria*, *Gaiella*, *Aciditer*, *Gp3*, *Massilia*, *Noviherbaspirillum*, *Ramlibacter*, *Flavisolibacter*, *Arthrobacter*, *Conexibacter*, *Adhaeribacter*, *Nitrospira*, *Denitratisoma*, *Blastococcus*, *Anaeromyxobacter*, and *Rubellimicrobium* were higher in T1 compared with T2. *Gp16*, *Ohtaekwangia*, *Steroidobacter*, *Phaselicystis*, *Lysobacter*, *Chondromyces*, *Phenylobacterium*, *Stakelama*, *Blastomonas*, *Gp10*, *Aquincola*, *Pimelobacter*, *Vasilyevaea*, *Novosphingobium*, *Iamia*, *Flavitalea*, *Jahnella*, *Croceicoccus*, *Ilumatobacter*, *Nocardioides*, *Terrimonas*, *Sphingobium*, *Anaeromyxobacter*, *Anderseniella*, *Blastobacter*, *Altererythrobacter*, and *Rubellimicrobium* were significantly higher in quadrat T2 compared with T3, whereas *Gemmatimonas*, *Candidatus Koribacter*, *Gaiella*, *Ktedonobacter*, *Gp3*, *Massilia*, *Flavisolibacter*, *Gp1*, *Conexibacter*, *Denitratisoma*, and *Blastococcus* were higher in T3 than T2.

## Discussion

The fragile ecosystems of karst area in Southwest China were formed on thick layers of carbonate rocks that provided material for karst rocky desertification. With porous carbonate rocks and concentrated rainy seasons, it is difficult to retain soil nutrients, and the soils usually have coarse texture (Table 1).

Previous studies suggested that N:P nutrient ratio in the aerial biomass could be a better index of deficiency of N or P than individual leaf nutrient concentrations^46^. The variability in foliar N:P ratios might differ greatly among plant species, and could vary by 50-fold in response to natural or experimental variation in supply of N and P^12^. Variation among species in the nutrient requirements might result in differences in N:P ratios to indicate N or P limitation to a particular species^7^,^47^. The N-limited species were often found at places where P controlled biomass production, and reverse was also true^8^. The N:P ratio greater than 16 usually indicated P limitation at the community level, and N:P ratio lower than 14 indicated N limitation^8^. In the present study, the foliar N:P ratio increased from 7.6 in T2 to 34 in T3 (Fig. 1), suggesting that nutrient limitation changed from N limitation in the karst area to P limitation in the unused agricultural field, likely reflecting the history of fertilization.

The properties of above- and belowground parts of the ecosystems are frequently correlated^19^,^20^. The amount and availability of soil nutrients are influenced by the interaction of terrain, climate and biological factors^48^. In the present study, foliar N:P ratios were positively correlated with soil total N in T2, and were negatively correlated with soil total P in T3 (Fig. 1A,B). The positive correlation between foliar N:P ratios and soil total N in the karst area (T2) indicated that the community foliar N:P ratios were mainly limited by soil N. Foliar N:P ratios in the unused agricultural field were negatively correlated with soil total P, but were not correlated with soil total N, indicating that pepino growth was mainly limited by soil P. The transition from N limitation in the karst area to P limitation in the unused agricultural field suggests that nitrogen-fixing plants could accelerate the recovery of barren karst ecosystems.

Soil enzymes are the important indicators of soil biochemistry and are linked with soil fertility and biological cycling^49^. Urease activity reflects the capacity of converting soil organic to inorganic nitrogen, catalase breaks down hydrogen peroxide to water and oxygen, and invertase hydrolyses sucrose into glucose and fructose^50^. With pepino planting, urease activity increased significantly, suggesting enhanced nitrogen cycling, but catalase activity decreased significantly, suggesting lower redox capacity.

The 16S rRNA gene sequencing results indicated an increasing diversity of bacteria after planting pepino in the karst area. Compared with T1 and T3 quadrats, T2 had a significantly higher percentage of *Proteobacteria* (1.3 and 1.4 times, respectively). *Proteobacteria* are one of the largest phyla of soil bacteria and include many nitrogen-fixing bacteria (e.g., rhizobia)^51^. The study of *Acidobacteria* began comparatively late, and their functions are still not clear, but they can grow in infertile soils, and therefore could be an indicator of barren soil environment^43^. T2 had a lower percentage of *Acidobacteria* than T1, which might indicate a better soil environment compared with T1.

*Sphingosinicella* is a relatively new genus, isolated from seawater. This genus has the capacity to degrade polyaromatic hydrocarbons to anthranilic acid^52^. Polycyclic aromatic compounds are widely distributed in nature and might arise from the diagenetic processes in the sediment^53^. *Sphingosinicella* was one of the dominant genera in T2, with significantly higher abundance than in T1, suggesting a potential for more acidic materials in the soil-forming processes in T2 than T1.

*Sphingomonas* is a typical genus of *Proteobacteria* and is widely distributed in nature. It is chemoheterotrophic, strictly aerobic, and it has been isolated from many different soil and water habitats, as well as from plant root systems. It can survive in environments limited in nutrients^54^. In addition, *Sphingomonas* was one of the most effective microbial groups in cleaning up toxic substances in soil^55^. Some *Sphingomonas* strains showed characteristics of denitrification and nitrogen fixation, and therefore may be involved in N cycling^54^. In our study, *Sphingomonas* was one of the dominant genera in T2, suggesting a potential for enhanced N cycling and maybe nitrogen fixation.

*Lysobacter* has higher content of G+C and higher biolytic activity than many other bacterial genera. *Lysobacter* was widely distributed in the rhizosphere soil of various plants^56^, and can secrete various antibiotics, enzymes and biologically-active materials to inhibit other bacteria and control plant diseases. In our study, *Lysobacter* was present in higher abundance in T1 and T2 compared with T3, which might indicate a soil environment with a lower plant disease burden in T1 and T2 than T3.

*Nocardioides* is a typical genus of *Proteobacteria* and is aerobic, mesophilic and prefers alkaline environments^57^. *Nocardioides* is not only widely distributed, but has applications in environmental management, such as *N. aromaticivorans* degrading furan truxene^58^ and *N. oleivorans* (isolated from oilfield in Germany) degrading crude oil^59^. In the present study, *Nocardioides* was significantly higher in T2 compared with T3, suggesting a higher capacity to decompose harmful hydrocarbons in T2 than T3.

This study increased our understanding of the diversity and structure of bacterial communities in karst soil and the important changes caused by pepino planting. Increased soil total nitrogen would enhance soil remediation processes in the karst ecosystem, suggesting the primary nitrogen limitation^60^.

## Additional Information

**How to cite this article**: Hu, J. *et al.* Pepino (*Solanum muricatum*) planting increased diversity and abundance of bacterial communities in karst area. *Sci. Rep.* **6**, 21938; doi: 10.1038/srep21938 (2016).

## Figures and Tables

**Table 1:** Soil basic properties and nutrient pools in three quadrats in the karst area.

Quadrat	Elevation (m)	pH	Soil particle distribution (%)			C_org_ (g·kg^−1^)	Total N (g·kg^−1^)	Total P (g·kg^−1^)
			d ≥ 0.02 mm	0.002 mm ≤ d < 0.02 mm	d < 0.002 mm			
T1	2169	7.5 ± 0.4a	77.5 ± 1.5a	12.1 ± 0.6b	10.4 ± 0.9b	4.3 ± 0.3b	0.9 ± 0.05ab	0.5 ± 0.10a
T2	2153	7.3 ± 0.5a	74.6 ± 1.2a	13.5 ± 1.2b	11.9 ± 0.1b	4.7 ± 0.1b	1.0 ± 0.02ab	0.5 ± 0.08a
T3	2135	6.8 ± 0.5b	24.5 ± 2.4b	20.0 ± 0.5a	55.5 ± 2.5a	10.3 ± 1.3a	1.3 ± 0.01a	0.4 ± 0.08b

Different letters in a single column indicate significant (p ≤ 0.05) differences. Means of three replicates ± standard error.

**Table 2:** Pearson correlation coefficients between C, N, P, K, Ca, C:N, C:P and N:P in the karst soils.

Element or ratios	C	N	P	K	Ca	C:N	C:P	N:P	Element or ratios
C		−0.31	0.15	−0.20	0.06	0.96**	0.73**	−0.36	C
N	0.21		0.49*	0.57*	−0.24	−0.54*	−0.52*	0.09	N
P	0.54	**0.039**		0.45	−0.36	−0.02	−0.47	−0.80**	P
K	0.44	**0.015**	0.06		−0.55*	−0.28	−0.24	−0.04	K
Ca	0.81	0.35	0.14	**0.019**		0.07	0.07	0.22	Ca
Mg	0.53	0.41	0.16	0.12	0.26	−0.25	−0.05	0.43	Mg
C:N	**<0.001**	**0.021**	0.95	0.26	0.78		0.83**	−0.30	C:N
C:P	**0.001**	**0.026**	0.05	0.33	0.79	**<0.001**		0.24	C:P
N:P	0.14	0.73	**<0.001**	0.88	0.39	0.22	0.33		N:P

^**^Significant at (p < 0.01); ^*^significant at (p ≤ 0.05).

The correlation coefficients are listed in the upper right triangle, and the corresponding *P* values in the lower left triangle.

**Table 3:** The urease, invertase and catalase activity of soil in the karst area.

Samples	Urease (mg·g^−1^·day^−1^)	Catalase (mL·g^−1^·30 min^−1^)	Invertase (mg·g^−1^·day^−1^)
S1	0.03 ± 0.01^c^	15.3 ± 0.52^a^	12.5 ± 0.28^b^
S2	0.12 ± 0.01^b^	12.9 ± 0.18^b^	11.7 ± 0.19^b^
RS2	0.14 ± 0.02^b^	9.63 ± 0.02^c^	10.48 ± 1.23^b^
S3	0.23 ± 0.02^a^	9.1 ± 0.15^c^	21.1 ± 1.10^a^
RS3	0.22 ± 0.01^a^	8.98 ± 0.52^c^	24.79 ± 2.17^a^

Different letters in a single column indicate significant (p ≤ 0.05) differences. Means of three replicates ± standard error.

**Table 4:** Comparison of the estimated operational taxonomic unit (OTU) richness and the diversity indices of the 16S rRNA gene libraries for clustering at 97% identity as obtained from the pyrosequecing analysis.

	Observed OTUs	Shannon index	Chao 1 index	Simpson’s diversity index (10^−2^)
S1	1759^c^	10.77^e^	1,575,917^c^	0.058^a^
S2	2712^c^	11.40^d^	3,686,725^bc^	0.037^b^
RS2	8372^a^	13.02^a^	3,5446,532^a^	0.012^d^
S3	4082^b^	11.95^c^	6,153,622^bc^	0.023^c^
RS3	4887^b^	12.25^b^	11,992,086^b^	0.021^c^

Means (n = 3). Different letters in a single column indicate significant (p ≤ 0.05) differences among the soils.

**Table 5:** The genera showing significant differences in percent abundance among the bulk and rhizosphere soils of three quadrats.

Taxon	Sample				
	S1	S2	S2	S3	RS3
*Gp6*	14.42 ± 0.68a	12.67 ± 0.71a	8.99 ± 1.25b	8.28 ± 0.57b	2.65 ± 0.45c
*Sphingomonas*	5.33 ± 0.02bc	8.41 ± 1.78a	6.69 ± 0.65ab	3.69 ± 0.07c	5.35 ± 0.29bc
*Gemmatimonas*	6.51 ± 0.51b	3.46 ± 0.23c	2.99 ± 0.07c	17.93 ± 0.35a	7.57 ± 0.44b
*Sphingosinicella*	2.38 ± 0.31c	6.20 ± 1.13a	4.23 ± 0.26b	2.56 ± 0.03bc	3.00 ± 0.09bc
*Gp4*	6.17 ± 0.55a	2.39 ± 0.30bc	2.28 ± 0.32c	3.48 ± 0.00b	1.35 ± 0.35c
*Dongia*	0.93 ± 0.05c	1.69 ± 0.14ab	2.12 ± 0.22a	0.90 ± 0.06c	1.62 ± 0.16b
*Gp16*	1.29 ± 0.17b	1.96 ± 0.04a	1.36 ± 0.10b	0.84 ± 0.01c	0.63 ± 0.07c
*Ohtaekwangia*	1.01 ± 0.09b	1.95 ± 0.06a	2.01 ± 0.09a	0.41 ± 0.06c	1.08 ± 0.11b
*Steroidobacter*	1.41 ± 0.08b	1.88 ± 0.20a	1.92 ± 0.21a	0.42 ± 0.04c	0.56 ± 0.08c
*Kofleria*	1.73 ± 0.13a	1.32 ± 0.05b	1.12 ± 0.05b	1.08 ± 0.01b	1.07 ± 0.18b
*Candidatus Koribacter*	0.35 ± 0.04b	0.38 ± 0.04b	0.47 ± 0.08b	3.31 ± 0.17a	2.99 ± 0.16a
*Gaiella*	1.44 ± 0.08b	0.86 ± 0.04c	0.34 ± 0.01d	1.84 ± 0.07a	1.45 ± 0.03b
*Phaselicystis*	1.05 ± 0.02b	1.47 ± 0.21a	1.15 ± 0.05b	0.57 ± 0.01c	0.47 ± 0.04c
*Aciditer*	1.36 ± 0.11a	1.09 ± 0.04b	0.79 ± 0.05c	0.59 ± 0.00c	0.67 ± 0.07c
*Ktedonobacter*	0.28 ± 0.06c	0.25 ± 0.04c	0.29 ± 0.06c	3.83 ± 0.37a	2.27 ± 0.11b
*Pseudolabrys*	0.53 ± 0.05c	0.74 ± 0.10bc	1.24 ± 0.13a	0.87 ± 0.00b	1.39 ± 0.10a
*Renibacterium*	1.09 ± 0.06a	0.82 ± 0.15ab	0.76 ± 0.03ab	0.73 ± 0.03b	0.95 ± 0.15ab
*Nitrosospira*	0.91 ± 0.08b	0.95 ± 0.12b	0.67 ± 0.03b	1.52 ± 0.06a	0.94 ± 0.12b
*Gp3*	1.26 ± 0.13b	0.64 ± 0.13c	0.34 ± 0.03c	2.34 ± 0.13a	1.51 ± 0.15b
*Lysobacter*	0.87 ± 0.26a	0.93 ± 0.12a	0.91 ± 0.15a	0.16 ± 0.14b	0.27 ± 0.12b
*Massilia*	2.36 ± 0.05a	0.42 ± 0.12c	0.35 ± 0.03c	1.04 ± 0.08b	0.8 ± 0.06b
*Noviherbaspirillum*	1.57 ± 0.11a	0.70 ± 0.08b	0.37 ± 0.05c	1.60 ± 0.04a	0.89 ± 0.04b
*Ramlibacter*	1.02 ± 0.02a	0.63 ± 0.07b	0.57 ± 0.09b	1.17 ± 0.14a	0.70 ± 0.01b
*Chondromyces*	0.41 ± 0.02bc	0.82 ± 0.13b	1.84 ± 0.25a	0.30 ± 0.03c	0.25 ± 0.05c
*Phenylobacterium*	0.50 ± 0.08b	0.93 ± 0.09a	0.99 ± 0.01a	0.45 ± 0.01b	0.60 ± 0.13b
*Stakelama*	0.13 ± 0.04d	0.71 ± 0.10b	1.10 ± 0.10a	0.38 ± 0.02c	0.46 ± 0.03c
*Saccharibacteria_genera_incertae_sedis*	0.30 ± 0.03c	0.56 ± 0.02bc	0.69 ± 0.11b	0.73 ± 0.10b	1.17 ± 0.10a
*Blastomonas*	0.24 ± 0.04c	0.81 ± 0.14b	1.06 ± 0.08a	0.18 ± 0.00c	0.35 ± 0.02c
*Gp10*	0.35 ± 0.11b	1.18 ± 0.15a	1.25 ± 0.05a	0.20 ± 0.01b	0.15 ± 0.11b
*Aquincola*	0.48 ± 0.02c	0.78 ± 0.08b	0.96 ± 0.08a	0.38 ± 0.01c	0.33 ± 0.05c
*Pimelobacter*	0.46 ± 0.02b	0.75 ± 0.13a	0.86 ± 0.07a	0.33 ± 0.05b	0.31 ± 0.02b
*Gp7*	0.80 ± 0.13a	0.53 ± 0.03b	0.91 ± 0.09a	0.47 ± 0.01b	0.36 ± 0.04b
*Longilinea*	0.57 ± 0.03ab	0.51 ± 0.04b	0.67 ± 0.06a	0.46 ± 0.01b	0.47 ± 0.06b
*Vasilyevaea*	0.17 ± 0.03b	0.84 ± 0.05a	1.08 ± 0.16a	0.18 ± 0.00b	0.31 ± 0.01b
*Flavisolibacter*	0.68 ± 0.11b	0.37 ± 0.04c	0.40 ± 0.07c	1.19 ± 0.08a	0.75 ± 0.12b
*Novosphingobium*	0.33 ± 0.04c	0.50 ± 0.03b	0.72 ± 0.04a	0.12 ± 0.01d	0.33 ± 0.02c
*Gp1*	0.08 ± 0.02c	0.02 ± 0.01c	0.07 ± 0.01c	1.64 ± 0.10b	2.33 ± 0.08a
*Iamia*	0.59 ± 0.01b	0.70 ± 0.04a	0.56 ± 0.03b	0.29 ± 0.02c	0.35 ± 0.01c
*Arthrobacter*	0.99 ± 0.01a	0.36 ± 0.12c	0.32 ± 0.04c	0.29 ± 0.03c	0.56 ± 0.05b
*Flavitalea*	0.84 ± 0.11a	0.62 ± 0.15ab	0.38 ± 0.02bc	0.28 ± 0.02c	0.18 ± 0.01c
*Jahnella*	0.80 ± 0.11a	0.62 ± 0.09ab	0.52 ± 0.01b	0.21 ± 0.01c	0.12 ± 0.03c
*Croceicoccus*	0.17 ± 0.01c	0.49 ± 0.11b	0.77 ± 0.04a	0.14 ± 0.01c	0.29 ± 0.01c
*Conexibacter*	0.92 ± 0.09a	0.30 ± 0.07c	0.27 ± 0.02c	0.79 ± 0.03ab	0.59 ± 0.12b
*Ilumatobacter*	0.40 ± 0.06c	0.95 ± 0.03a	0.66 ± 0.04b	0.11 ± 0.01d	0.13 ± 0.02d
*Adhaeribacter*	1.85 ± 0.13a	0.40 ± 0.04b	0.23 ± 0.02bc	0.05 ± 0.01c	0.03 ± 0.01c
*Blastocatella*	0.64 ± 0.04a	0.39 ± 0.06bc	0.54 ± 0.02ab	0.52 ± 0.03abc	0.35 ± 0.09c
*Nocardioides*	0.44 ± 0.04a	0.44 ± 0.04a	0.54 ± 0.03a	0.21 ± 0.02b	0.23 ± 0.03b
*Terrimonas*	0.49 ± 0.05a	0.51 ± 0.06a	0.58 ± 0.02a	0.29 ± 0.02b	0.21 ± 0.03b
*Rhizobium*	0.07 ± 0.03c	0.49 ± 0.11a	0.30 ± 0.04b	0.04 ± 0.00c	0.50 ± 0.03a
*Nitrospira*	0.78 ± 0.07a	0.50 ± 0.05b	0.14 ± 0.03c	0.91 ± 0.05a	0.42 ± 0.03b
*Sphingobium*	0.04 ± 0.01d	0.37 ± 0.05b	0.89 ± 0.08a	0.03 ± 0.00d	0.18 ± 0.01c
*Denitratisoma*	0.41 ± 0.06bc	0.28 ± 0.05cd	0.17 ± 0.02d	1.29 ± 0.07a	0.52 ± 0.01b
*Ottowia*	0.57 ± 0.02a	0.49 ± 0.03a	0.34 ± 0.06b	0.38 ± 0.01b	0.27 ± 0.03b
*Gp17*	0.66 ± 0.07a	0.54 ± 0.08a	0.24 ± 0.03b	0.13 ± 0.00b	0.09 ± 0.01b
*Parasegetibacter*	0.62 ± 0.11a	0.76 ± 0.13a	0.28 ± 0.01b	0.08 ± 0.00b	0.08 ± 0.02b
*Blastococcus*	0.46 ± 0.04b	0.22 ± 0.02c	0.21 ± 0.02c	0.90 ± 0.03a	0.47 ± 0.07b
*Anaeromyxobacter*	0.85 ± 0.03a	0.40 ± 0.05b	0.34 ± 0.03bc	0.22 ± 0.02cd	0.16 ± 0.09d
*Nitrobacter*	0.19 ± 0.10c	0.37 ± 0.04b	0.25 ± 0.04bc	0.33 ± 0.01bc	0.61 ± 0.02a
*Anderseniella*	0.12 ± 0.01b	0.59 ± 0.11a	0.54 ± 0.06a	0.15 ± 0.00b	0.16 ± 0.02b
*Blastobacter*	0.17 ± 0.06bc	0.37 ± 0.04a	0.43 ± 0.05a	0.06 ± 0.01c	0.21 ± 0.04b
*Altererythrobacter*	0.26 ± 0.03b	0.57 ± 0.09a	0.44 ± 0.05a	0.10 ± 0.01c	0.08 ± 0.00c
*Rubellimicrobium*	0.63 ± 0.10a	0.39 ± 0.04b	0.46 ± 0.01b	0.03 ± 0.01c	0.02 ± 0.01c
*Devosia*	0.11 ± 0.02cd	0.39 ± 0.10b	0.61 ± 0.06a	0.03 ± 0.01d	0.22 ± 0.01bc

Means ± standard error (n = 3); means followed by different letters in a row (one-way ANOVA) are significantly different at P ≤ 0.05.
